# Estimation of Inbreeding Depression From Overdominant Loci Using Molecular Markers

**DOI:** 10.1111/eva.70085

**Published:** 2025-03-13

**Authors:** Inés González‐Castellano, Pilar Ordás, Armando Caballero

**Affiliations:** ^1^ Centro de Investigación Mariña, Universidade de Vigo Vigo Spain; ^2^ Universidade da Coruña A Coruña Spain

**Keywords:** Coancestry, deleterious recessive mutations, genetic drift, identity by descent, inbreeding load, runs of homozygosity

## Abstract

Inbreeding depression is a highly relevant universal phenomenon in population and conservation genetics since it leads to a decline in the fitness of individuals. This phenomenon is due to the homozygous expression of alleles whose effects are hidden in heterozygotes (inbreeding load). The rate of inbreeding depression for quantitative traits can be quantified if the coefficient of inbreeding (*F*) of individuals is known. This coefficient can be estimated from pedigrees or from the information of molecular markers, such as SNPs, using measures of homozygosity of individual markers or runs of homozygosity (ROH) across the genome. Several studies have investigated the accuracy of different *F* measures to estimate inbreeding depression, but always assuming that this is only due to recessive or partially recessive deleterious mutations. It is possible, though, that part of the inbreeding depression is due to variants with overdominant gene action (heterozygote advantage). In this study, we carried out computer simulations to assess the impact of overdominance on the estimation of inbreeding depression based on different measures of *F*. The results indicate that the estimators based on ROH provide the most robust estimates of inbreeding depression when this is due to overdominant loci. The estimators that use measures of homozygosity from individual markers may provide estimates with substantial biases, depending on whether or not low‐frequency alleles are discarded in the analyses; but among these SNP‐by‐SNP measures, those based on the correlation between uniting gametes are generally the most reliable.

## 1 Introduction

The decrease in individual fitness resulting from inbreeding, known as inbreeding depression, is a key topic for understanding the evolution of populations and for the management and conservation of both wild and domestic species (Wright 1977; Thornhill 1993; Lynch and Walsh 1998; Charlesworth and Willis 2009; Caballero 2020). Inbreeding depression is a major concern for small, endangered populations, as it can ultimately lead them to extinction (O'Grady et al. 2006; Allendorf et al. 2013). Although inbreeding appears to universally reduce fitness, its magnitude and specific effects are highly variable depending on the genetic makeup of species or populations and how genotypes interact with the environment (Hedrick and Kalinowski 2000). Two hypotheses have been proposed regarding the genetic causes underlying inbreeding depression: the partial dominance hypothesis and the overdominance hypothesis (Charlesworth and Charlesworth 1999; Keller and Waller 2002; Charlesworth and Willis 2009; Hasselgren and Norén 2019). The partial dominance hypothesis (Davenport 1908) holds that inbreeding depression results from the expression of recessive or partially recessive deleterious alleles present at low frequencies in populations, with their effects being fully or partially masked by dominant alleles when present in heterozygotes (Morton et al. 1956). As inbreeding increases homozygosity, these deleterious mutations become expressed in the population, leading to a decreased fitness (Davenport 1908; Charlesworth and Willis 2009). The second hypothesis is overdominance (East 1908; Shull 1908), a term referring to the genetic condition of heterozygote advantage, implying that a genetic polymorphism is maintained at a locus through balancing selection (Fisher 1922; Charlesworth and Willis 2009). This hypothesis proposes that the advantage of heterozygotes over either of the homozygotes is what reduces population fitness as heterozygosity decreases due to increased inbreeding. Thus, inbreeding depression is the consequence of the expression of the inbreeding load, which is expressed when homozygous frequencies increase because of higher inbreeding (Morton et al. 1956).

Despite the general view of a major role of dominance in explaining inbreeding depression (Charlesworth and Charlesworth 1999; Roff 2002; Charlesworth and Willis 2009; Hedrick 2012; Yang et al. 2017), the contribution of overdominance cannot be neglected. There is evidence of genes exhibiting overdominance (Stuber et al. 1992; Luo et al. 2001; Charlesworth 2006; Charlesworth and Charlesworth 2010; Sellis et al. 2011; Hedrick 2012; Fijarczyk and Babik 2015), and gene expression studies in inbred lines of *Drosophila* suggest a small proportion of genes under overdominance (Gibson et al. 2004; Hughes et al. 2006; Ayroles et al. 2009). Thurman and Barrett (2016) found that about 2% of estimated selection coefficients in a meta‐analysis of the literature from natural populations of multiple taxa could be overdominant. There are also studies suggesting an excess of genetic variance for fitness traits in Drosophila that could not be explained by mutation‐selection balance alone (Charlesworth 2015; Sharp and Agrawal 2018). Moreover, about 0.5% of human genes show signs of balancing selection (Andrés et al. 2009). This may occur through antagonistic pleiotropy arising from a negative relationship between reproduction and lifespan (Lemaître et al. 2015; Brown and Kelly 2018), from opposing effects on early‐onset and late‐onset diseases (Rodríguez et al. 2017), or from genetic variants that have been favored in the past that can now be deleterious for some traits in the current environment (Corbett et al. 2018). Even if the number of overdominant loci is just a few, these could make a very large contribution to the inbreeding load (Kimura and Ohta 1971).

The rate of inbreeding depression is generally quantified by the slope of the linear regression of individual phenotypic values on their coefficient of inbreeding (*F*; the probability of alleles being identical by descent) (Lynch and Walsh 1998). The coefficient of inbreeding of an individual has traditionally been studied using pedigrees (Wright 1969). However, in practice, genealogical information is difficult to obtain, at least for wild species, and is commonly used to calculate only recent inbreeding (usually up to a few generations back). Furthermore, genealogical *F* represents an expected value of the proportion of the genome that is homozygous by descent, but there is considerable variation around this estimate due to the stochastic nature of recombination (Hill and Weir 2011; Keller et al. 2011). Nevertheless, an individual's level of inbreeding can also be inferred from genome‐wide homozygosity, mainly using high‐density molecular markers such as single nucleotide polymorphisms (SNPs) (Keller et al. 2011; Curik et al. 2014; Wang 2016). The statistical power to detect inbreeding depression using SNP data depends on the variation in individual inbreeding within the population, the magnitude of inbreeding depression, and the sample size (Keller et al. 2011).

Various methods exist to obtain genomic estimates of inbreeding from individual SNPs, whether based on maximum likelihood, the diagonal elements of a genomic relationship matrix, homozygosity measures, or genotypic correlations (e.g., Ritland 1996; Purcell et al. 2007; Wang 2007; Van Raden 2008; Szulkin et al. 2010; Yang et al. 2011; Bjelland et al. 2013). Methods are also based on the proportion of the genome comprised of “runs of homozygosity” (ROH) (McQuillan et al. 2008; Ferenčaković et al. 2017; Ceballos et al. 2018) or segments of homozygosity identical by descent (Druet and Gautier 2017), which are highly correlated with each other (Alemu et al. 2021).

Multiple empirical comparisons have been made between estimates of inbreeding depression using different measures of inbreeding (Santure et al. 2010; Keller et al. 2011; Bjelland et al. 2013; Pryce et al. 2014; Saura et al. 2015; Zhang et al. 2015; Kardos et al. 2016; Goudet et al. 2018; Alemu et al. 2021; Villanueva et al. 2021; Ojeda‐Marín et al. 2023; Pérez‐Pereira, Quesada, et al. 2023). These analyses with empirical data are very insightful, but the true inbreeding depression is unknown, making it challenging to draw conclusions about which genomic measure of *F* provides more accurate estimates. Computer simulations with known parameters, however, allow for the evaluation of the accuracy of some *F* measures in estimating inbreeding depression (e.g., Keller et al. 2011; Yengo et al. 2017; Nietlisbach et al. 2019; Caballero et al. 2021, 2022; Lavanchy et al. 2024). Several studies have indicated that the power and accuracy of different *F* measures for estimating inbreeding depression depend on the scenarios considered, with those based on ROH or the correlation between uniting gametes being the most reliable (Yengo et al. 2017; Nietlisbach et al. 2019; Caballero et al. 2021, 2022; Lavanchy et al. 2024). To date, only a single study has considered the impact of overdominance in estimating inbreeding depression (Curik et al. 2001). This study was restricted to a two‐locus model comparing the estimates of inbreeding depression obtained from pedigree inbreeding and from molecular autozygosity. The aim of the present study is to evaluate, through computer simulations, a range of molecular measures of inbreeding in order to assess which one offers a more accurate estimation of inbreeding depression when this is due not only to partially recessive deleterious mutations but also to overdominant loci.

## 2 Methods

### 2.1 Simulation Procedure and Genetic Parameters

The programme SLiM3 (Haller and Messer 2019) was used to carry out time‐forward individual‐based simulations of a diploid monoecious population of constant size with *N* = 1000 or 10,000 individuals run for 10,000 discrete generations under random mating and the influence of mutation, recombination, selection, and genetic drift. The simulation software performs a model of fecundity selection, such that all offspring survive to maturity, and fitness specifies the probability that an individual will be chosen as a parent in the next generation. Higher fitness individuals thus have a larger expected progeny number, but fitness is relative because the population size is held constant in the simulations. In order to consider realistic recombination rates, the simulated genome sequences corresponded to 12 different regions of the human genome, each of about 90 Mb (Figure S1), and the corresponding genetic maps were included in the SLiM input to simulate the recombination events.

For each of the sequences simulated, the global mutation rate was assumed to be either 1.6 × 10^−8^ (for simulations with *N* = 1000 individuals) or 1.6 × 10^−9^ (for *N* = 10,000) per nucleotide per generation, in order to obtain a sufficiently large number of neutral SNPs (about 40,000 in each simulation). The genotypic fitness values for the wild‐type homozygote, the heterozygote, and the mutant homozygote were 1, 1 + *sh*, and 1 + *s*, respectively, where *s* is the selection coefficient and *h* the dominance coefficient. Neutral (*s* = 0), deleterious (*s* < 0), and overdominant (*s* > 0) mutations occurred randomly throughout the sequences. Mutational parameters for deleterious mutations followed those considered by Pérez‐Pereira, López‐Cortegano, et al. (2023). The selection coefficient *s* was obtained from a gamma distribution with shape parameter *β* = 0.33 and mean effect $\bar{s}$ = −0.2. Mutations with values of *s* less than −1 were assigned a value of −1. All mutations with *s* < −0.9 were considered sterile alleles. The dominance coefficient *h* of mutations was obtained from a uniform distribution between 0 and *e* ^ks^ (Caballero and Keightley 1994), where *k* is a constant that yields an average dominance coefficient of $\bar{h}$ = 0.283. The rate of deleterious mutations (see Table 1) was chosen so as to generate an inbreeding load of about one sterile equivalent, which is defined as a group of mutant genes of such number that on average, they would cause one complete sterility (Morton 1960; Chung 1962). The deleterious mutational parameters considered are in agreement with the estimates obtained from mutation–accumulation experiments for a range of higher eukaryotic species reviewed by Halligan and Keightley (2009): median *U* = 0.04 and mean $\bar{s}$ = −0.22. The assumed average dominance coefficient is concordant with the range of estimates obtained in different studies (García‐Dorado and Caballero 2000; Agrawal and Whitlock 2011; Manna et al. 2011; Caballero 2020, 158).

**TABLE 1:** Mutational parameters assumed in the simulations.

	*N* = 1000	*N* = 10,000
Nucleotide mutation rate	1.6 × 10^−8^	1.6 × 10^−9^
Neutral mutations		
*U*	1.37	0.12
*s*	0	
Deleterious mutations		
*U*	0.07	0.03
$\bar{s}$	−0.2	
*β*	0.33	
$\bar{h}$	0.283	
Overdominant mutations		
*U* (low overdominance)	0.00015	0.000015
*U* (high overdominance)	0.00075	0.000075
*s*	0.02	
*h*	1.5	

*Note:* Simulations were run assuming neutral and deleterious mutations or also adding overdominant mutations at a low or high mutation rate.

Abbreviations: *h*, dominance coefficient of mutations (the upper bar indicates average value); *N*, population size considered; *s*, selection coefficient of mutations (the upper bar indicates average value); *U*, haploid genomic mutation rate per generation; *β*, shape parameter for the gamma distribution of deleterious mutational effects.

For overdominant mutations, the selection coefficient and dominance coefficient were set at *s* = 0.02 and *h* = 1.5, respectively, such that the heterozygote had a higher fitness than both homozygotes. Models without overdominance and with overdominant mutations occurring at two different rates were considered, hereafter referred to as low and high overdominance, respectively (Table 1). A case was also run including only overdominant mutations. A multiplicative model for fitness was assumed across the genome.

### 2.2 Measures of the Inbreeding Coefficient

From the last generation of each simulation, files with the genotypic values of all neutral segregating SNPs (i.e., deleterious and overdominant mutations were excluded) were obtained for all individuals of the population. Estimates of individual inbreeding coefficients (*F*) were obtained assuming no minor allele frequency (MAF) pruning or with a pruning for MAF = 0.05. We considered the measures of the coefficient of inbreeding investigated by Caballero et al. (2022), which were calculated as follows:

- $F_{\mathrm{VR}1}=\frac{\sum_{k=1}^{S}{\left(x_{k}-2p_{k}\right)}^{2}}{\sum_{k=1}^{S}2p_{k}\left(1-p_{k}\right)}-1$, where *S* is the total number of markers, $x_{k}$ is the number of minor alleles of marker *k* (i.e., 0, 1, or 2 copies), and $p_{k}$ is the frequency of the minor allele. This estimator was proposed by Van Raden (2008) and it is based on the variance of additive genetic values.
- $F_{\mathrm{VR}2}=\frac{1}{S}\sum_{k=1}^{S}\left(\frac{{\left(x_{k}-2p_{k}\right)}^{2}}{2p_{k}\left(1-p_{k}\right)}-1\right)$, a metric similar to *F*_*VR*1_ but with a different weighting, proposed by Leutenegger et al. (2003) and Amin et al. (2007), as cited by Van Raden (2008), who referred to it as “method 2”, by which it is commonly known in the Literature. This method was shown by Van Raden (2008) to have a lower correlation with pedigree inbreeding than *F*_*VR*1_.
- $F_{\mathrm{LH}1}=1-\frac{\sum_{k=1}^{S}x_{k}\left(2-x_{k}\right)}{\sum_{k=1}^{S}2p_{k}\left(1-p_{k}\right)}$, a metric initially proposed by Li and Horvitz (1953), which gives the deviation of the observed proportion of homozygotes from the expected value under Hardy–Weinberg equilibrium.
- $F_{\mathrm{LH}2}=1-\frac{1}{S}\sum_{k=1}^{S}\left(\frac{x_{k}\left(2-x_{k}\right)}{2p_{k}\left(1-p_{k}\right)}\right)$, which is analogous to *F*_*LH*1_ with a different weighting.
- $F_{\mathrm{NJ}}=1-\frac{1}{S}\sum_{k=1}^{S}x_{k}\left(2-x_{k}\right)$, the proportion of homozygous SNPs (Nejati‐Javaremi et al. 1997), which has a correlation of one with *F*_*LH*1_.
- $F_{\mathrm{YA}2}=\frac{1}{S}\sum_{k=1}^{S}\frac{x_{k}^{2}-\left(1+2p_{k}\right)x_{k}+2p_{k}^{2}}{2p_{k}\left(1-p_{k}\right)}$, which was proposed by Yang et al. (2011), and it is based on the correlation between uniting gametes, such that homozygous genotypes are weighted by the inverse of their allele frequencies.
- $F_{\mathrm{YA}1}=\frac{\sum_{k=1}^{S}x_{k}^{2}-\left(1+2p_{k}\right)x_{k}+2p_{k}^{2}}{\sum_{k=1}^{S}2p_{k}\left(1-p_{k}\right)}$, an analogous estimator to that of Yang et al. (2011) (*F*_*YA*2_) but considering the summation of terms separately in the numerator and denominator, as recommended by Zhang et al. (2022) and applied in simulations by Caballero et al. (2022) in panmictic populations and Lavanchy et al. (2024) in subdivided populations. Note that the estimators with subscript 1 involve a ratio of summations from markers, whereas those with subscript 2 involve the summation of ratios.

These estimators are based on genetic drift or on homozygosity, with both approaches being equivalent (Cockerham 1969). The relationships between them can be seen in the supplementary appendices of Caballero et al. (2021, 2022). Estimates for all the above *F* measures were obtained with an in‐house C program and were verified to match those obtained by PLINK (Purcell et al. 2007) and GCTA (Yang et al. 2011). Using these programs, *F*_*VR*1_, *F*_*LH*2_, *F*_*YA*2_, and *F*_*LH*1_ can be obtained with the so named *F* ^I^, *F* ^II^, *F* ^III^, and *F*_*HOM*_, respectively, and *F*_*VR*1_ can be obtained from the diagonal of the genomic relationship matrix obtained by GCTA with the option –*make‐grm‐alg 1*.

All the above molecular measures of inbreeding are expected to provide estimates of identity by descent with reference to a given base population of noninbred and unrelated individuals, provided the allele frequencies (*p*_*k*_) of all SNPs segregating at that base population are considered in the equations (Van Raden 2008; Wang 2014). In general, however, these are not known, and those of the current generation are used. We assume here this latter scenario, and current allele frequencies are assumed. Estimates from *F*_*VR*1‐2_, *F*_*LH*1‐2_, and *F*_*YA*1‐2_ then provide correlations between alleles carried by individuals or deviations from Hardy–Weinberg proportions in the current generation. Thus, they take positive or negative values, generally implying an excess or deficit of homozygotes. In contrast, *F*_*NJ*_, which provides direct measures of molecular homozygosity, only takes positive values from zero to one, as they include the proportion of homozygous SNPs in the genome. The nomenclature used here for the different methods may be different in other studies. The correspondence between some of the different acronyms used in the literature is shown in Table 1 of Villanueva et al. (2021).

Finally, we considered estimates of inbreeding from runs of homozygosity (ROH):

$F_{R}=\frac{\sum L_{\mathrm{ROH}}}{L}$, where Σ*L*_*ROH*_ is the sum of the lengths of all ROH that cover the genome of an individual, and *L* is the genome length (Broman and Weber 1999; McQuillan et al. 2008). We used the software PLINK1.9 with the default options: a minimum of 100 SNPs, at least 1 SNP per 50 kb, a scanning window of 50 SNPs, and two SNPs in the same ROH cannot be more than 1000 kb apart. ROH longer than 0.1 Mb (*F*_*R*01_), 1 Mb (*F*_*R*1_), or 5 Mb (*F*_*R*5_) were considered after removing highly linked SNPs (*r* ^2^ > 0.9) with the –indep‐pairwise 50 5 0.9 option, as recommended by Howrigan et al. (2011). These coefficients of inbreeding are expected to take positive values from zero to one, as they include the proportion of genomic regions that are homozygous.

### 2.3 Expected and Estimated Rates of Inbreeding Depression

The expected rate of inbreeding depression was quantified by the inbreeding load (*B*), which can be obtained as the sum of 2*dpq* values for all segregating mutations with an effect on fitness (Morton et al. 1956), where *p* and *q* are the frequencies of the wild‐type and mutant alleles, respectively, and *d* = *s* (*h* – ½) for deleterious and sterile alleles, or *d* = *s* (*h* − ½)/(1 + *sh*) for overdominant alleles. The estimated rate of inbreeding depression was obtained as the slope of the linear regression of the logarithm of individual fitness values on their *F* measures (Morton et al. 1956; Lynch and Walsh 1998). This is the standard method when the fitnesses of individuals follow a multiplicative fitness model and do not take zero values, for which logarithms would be undefined. Nietlisbach et al. (2019) showed that this logarithmic regression method is very accurate and only slightly biased for very high inbreeding loads (*B* > 20). The simulation model carried out with SLiM implies fecundity selection and a very low frequency of sterile alleles. Thus, because the population sizes considered were large (*N* = 1000 or 10,000 individuals), no homozygotes for purely sterile alleles (*s* = −1) appeared in any simulation. If individuals with zero fitness were to appear, such as for mortality traits (e.g., binary variables representing dead or alive), other alternative methods for inbreeding depression estimation should be considered, as discussed by Nietlisbach et al. (2019).

The estimated rate of inbreeding depression was compared with the expected inbreeding load by calculating the root mean squared error (RMSE), a combined measure of accuracy and precision in estimation. In addition to calculating the total inbreeding load, the proportion of this due to deleterious, sterile, and overdominant alleles was also calculated. The number of segregating neutral, deleterious, sterile, and overdominant loci, as well as their average allele frequency was also obtained. For each overdominance scenario (i.e., no overdominance, low overdominance, or high overdominance), the entire simulation process was repeated 20 times for each genomic region considered. Replicates and genomic regions were averaged for each case, and the standard error of means was obtained.

## 3 Results

Table 2 displays the average number of segregating loci for each type of mutation, along with their standard errors, for the simulations with *N* = 1000 individuals (the case for *N* = 10,000 is shown in Table S1). The number of neutral, deleterious, and sterile SNPs was very similar for the three scenarios (no overdominance and low and high overdominance), whereas the number of overdominant SNPs increased with the increase in the overdominance mutation rate, as expected. However, the mean frequency of neutral, deleterious, and sterile alleles increased with the increase in the overdominant mutation rate. The frequency of overdominant alleles approached the expected equilibrium frequency (0.75) with low overdominance (Low OD) mutation rate but was a bit lower with high overdominance (High OD) mutation rate. The lower part of the table shows the average inbreeding load (*B*) and the contribution of the different types of mutations to it. Note that the inbreeding load from deleterious and sterile mutations sensibly increased with overdominance, because of the increase in allele frequencies mentioned above.

**TABLE 2:** Average number of segregating loci and mean allelic frequency of neutral, deleterious, sterile, and overdominant mutations and inbreeding load attributable to deleterious (*B*_*del*_), sterile (*B*_*ste*_), overdominant (*B*_*o*_), or all (*B*) mutations.

Model	Neutral	Deleterious	Sterile	Overdominant
No. of SNPs				
No OD	41576.1 ± 484.5	1247.02 ± 14.52	58.90 ± 0.88	—
Low OD	42741.1 ± 494.6	1259.80 ± 14.69	57.81 ± 0.83	114.09 ± 1.44
High OD	41001.9 ± 452.3	1185.13 ± 13.10	53.40 ± 0.75	460.27 ± 4.99
Freq. of SNPs				
No OD	0.111 ± 0.000	0.034 ± 0.000	0.005 ± 0.000	—
Low OD	0.125 ± 0.000	0.041 ± 0.000	0.005 ± 0.000	0.711 ± 0.001
High OD	0.150 ± 0.001	0.057 ± 0.000	0.006 ± 0.000	0.665 ± 0.001

Inbreeding load	*B*	*B*_*del*_	*B*_*ste*_	*B*_*o*_
No OD	0.939 ± 0.012	0.653 ± 0.009	0.286 ± 0.005	—
Low OD	1.756 ± 0.022	0.697 ± 0.009	0.299 ± 0.006	0.759 ± 0.010
High OD	4.380 ± 0.047	0.779 ± 0.013	0.324 ± 0.007	3.276 ± 0.035

*Note:* Results refer to a population of size *N* = 1000 for scenarios with partial recessive deleterious mutations without overdominance (No OD), or also adding a low rate (Low OD), or a high rate (High OD), of overdominant mutations. The results are the averages of simulations for 12 chromosomal segments and 20 replicates each, and its corresponding standard errors.

The estimated inbreeding coefficients of individuals averaged across the whole population are shown in Table S2. The observed effect of overdominance was an increase in the average inbreeding coefficient, as deduced from long ROH (> 1 or 5 Mb), which are likely to involve homozygosity identical by descent and have relatively low standard errors.

**FIGURE 1:**
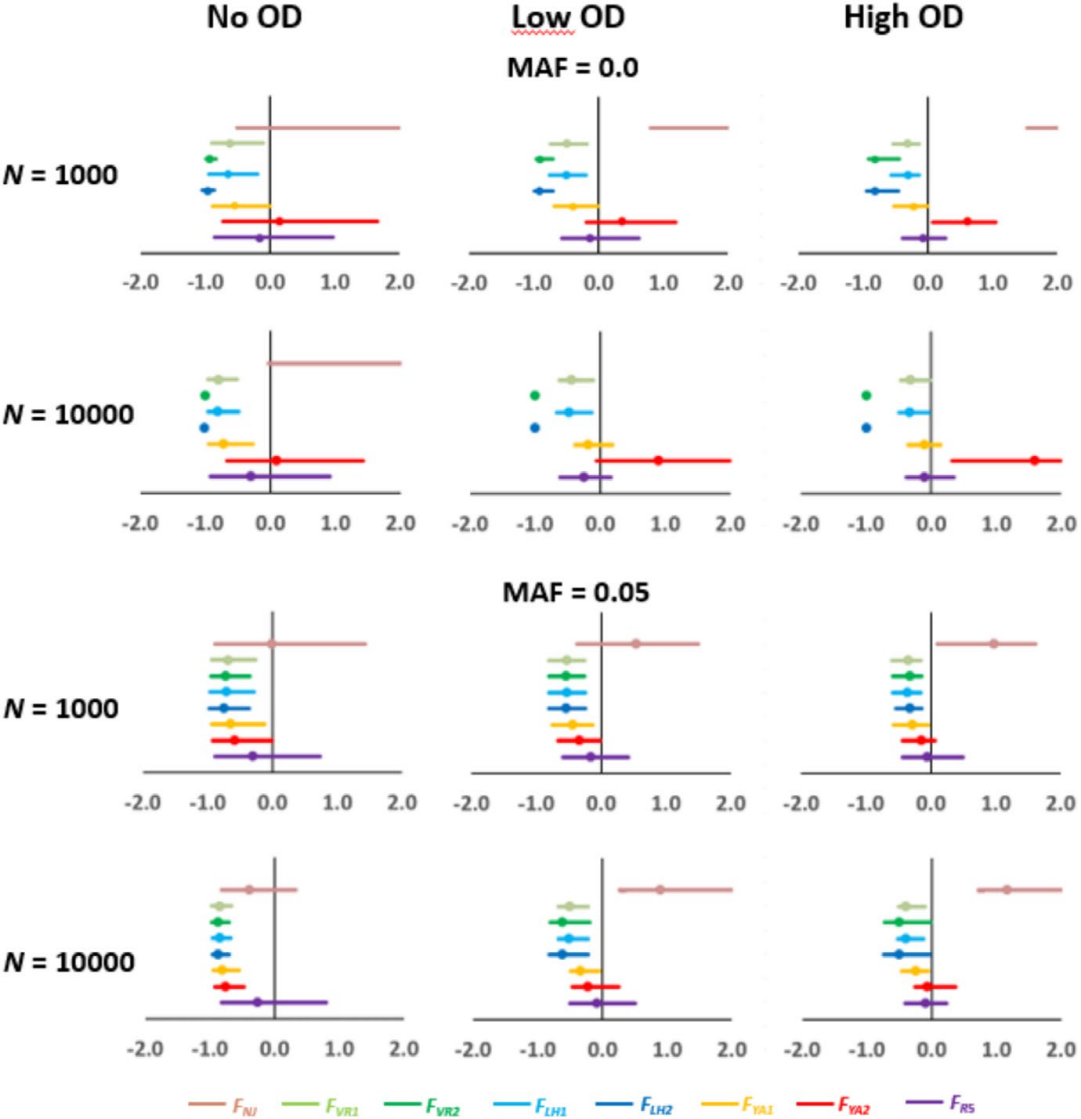
Proportional deviation of the estimates of inbreeding depression (ID) obtained with different measures of the inbreeding coefficient with marker data (see text), with respect to the true simulated ID value. Simulations assume different population sizes (*N* = 1000 or 10,000 individuals). The scenarios assume partial recessive deleterious mutations without overdominance (No OD), or also adding a low rate (Low OD), or a high rate (High OD), of overdominant mutations. The results are the averages of simulations for 12 chromosomal segments and 20 replicates each. The dot is the mean deviation and the bar indicates the 95% of the distribution of simulated replicates. See text for details on the simulation parameters.

**FIGURE 2:**
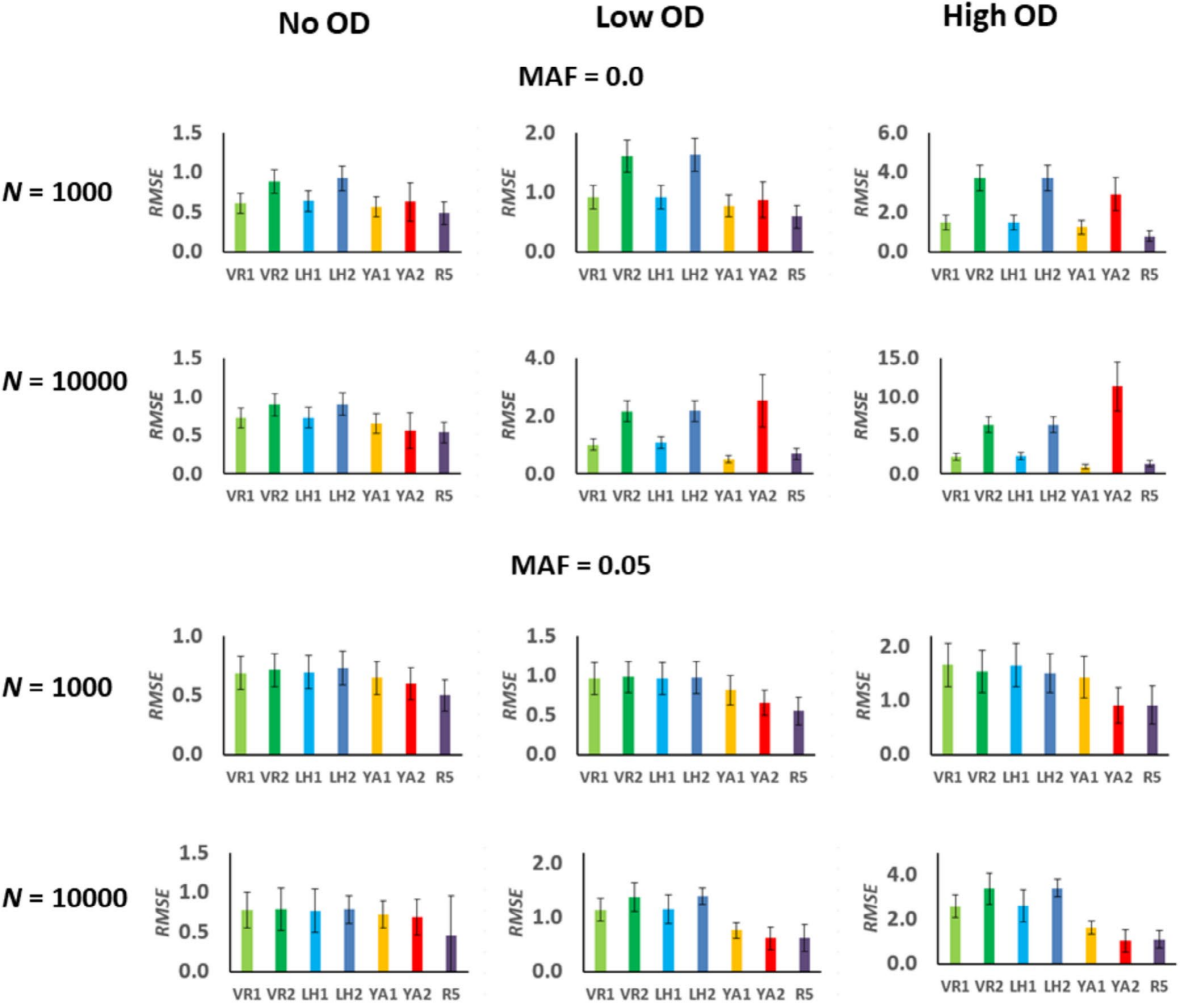
Root mean square error (RMSE) of estimates of inbreeding depression obtained from the different measures of the individual inbreeding coefficient (see text; only the subscripts of the estimators are shown for clarity). Simulations assume different population sizes (*N* = 1000 or 10,000 individuals). The scenarios assume partial recessive deleterious mutations without overdominance (No OD), or also adding a low rate (Low OD), or a high rate (High OD), of overdominant mutations. The results are the averages of simulations for 12 chromosomal segments and 20 replicates each, and bars indicate one standard error of the mean. See text for details on the simulation parameters.

Figure 1 shows the proportional deviations of the estimated inbreeding depression rate (ID) obtained with different molecular measures of *F* with respect to the true simulated inbreeding load (*B*). The bars exclude 2.5% of the extreme deviations at each side and the dot is the arithmetic mean of the deviations. Thus, a value of, say +1, implies an overestimation of ID by 100% and a value of −1 an underestimation of ID by 100%. The average root mean square error (RMSE) of estimates are given in Figure 2. The estimates based on molecular homozygosity (*F*_*NJ*_) were generally very biased upward, and the bars in Figure 1 were often overlapping, or even outside of the maximum deviation of +2 presented, such as for the scenarios with *N* = 10,000 individuals with no MAF pruning and overdominance. Although *F*_*NJ*_ and *F*_*LH*1_ are perfectly correlated, the scales of them are different, providing very different estimates of ID. In particular, the estimate from *F*_*LH*1_ equals that from *F*_*NJ*_ multiplied by the expected frequency of heterozygotes (see Supporting Information). Thus, *F*_*NJ*_ estimates are not further discussed.

First consider the estimates obtained with no overdominance and no MAF pruning, which are those considered in previous simulation studies. The most accurate and centered estimators were *F*_*YA*2_ and *F*_*R*5_, the latter referring to ROH fragments longer than 5 Mb. Results from ROH > 0.1 or 1 Mb were generally less accurate (downwardly biased) than those for ROH > 5 Mb (Figure S2). Estimates from *F*_*VR*2_ and *F*_*LH*2_ were heavily downwardly biased. When an MAF pruning was carried out, the general outcome was a reduction of the estimates of inbreeding depression, except for *F*_*VR*2_ and *F*_*LH*2_, whose estimates became higher, although still being underestimations. Estimates from *F*_*YA*2_ then became underestimates with MAF pruning but with more precision. However, those from *F*_*R*5_ did not change much with MAF pruning.

With overdominance and no MAF pruning, the estimates from *F*_*YA*2_ became severe overestimations, whereas those from *F*_*YA*1_ became more accurate. When MAF pruning was applied, both *F*_*YA*1_ and *F*_*YA*2_ increased in precision. Overall, the estimates from ROH were the most accurate and robust across scenarios, showing generally the lowest RMSE (Figure 2).

**FIGURE 3:**
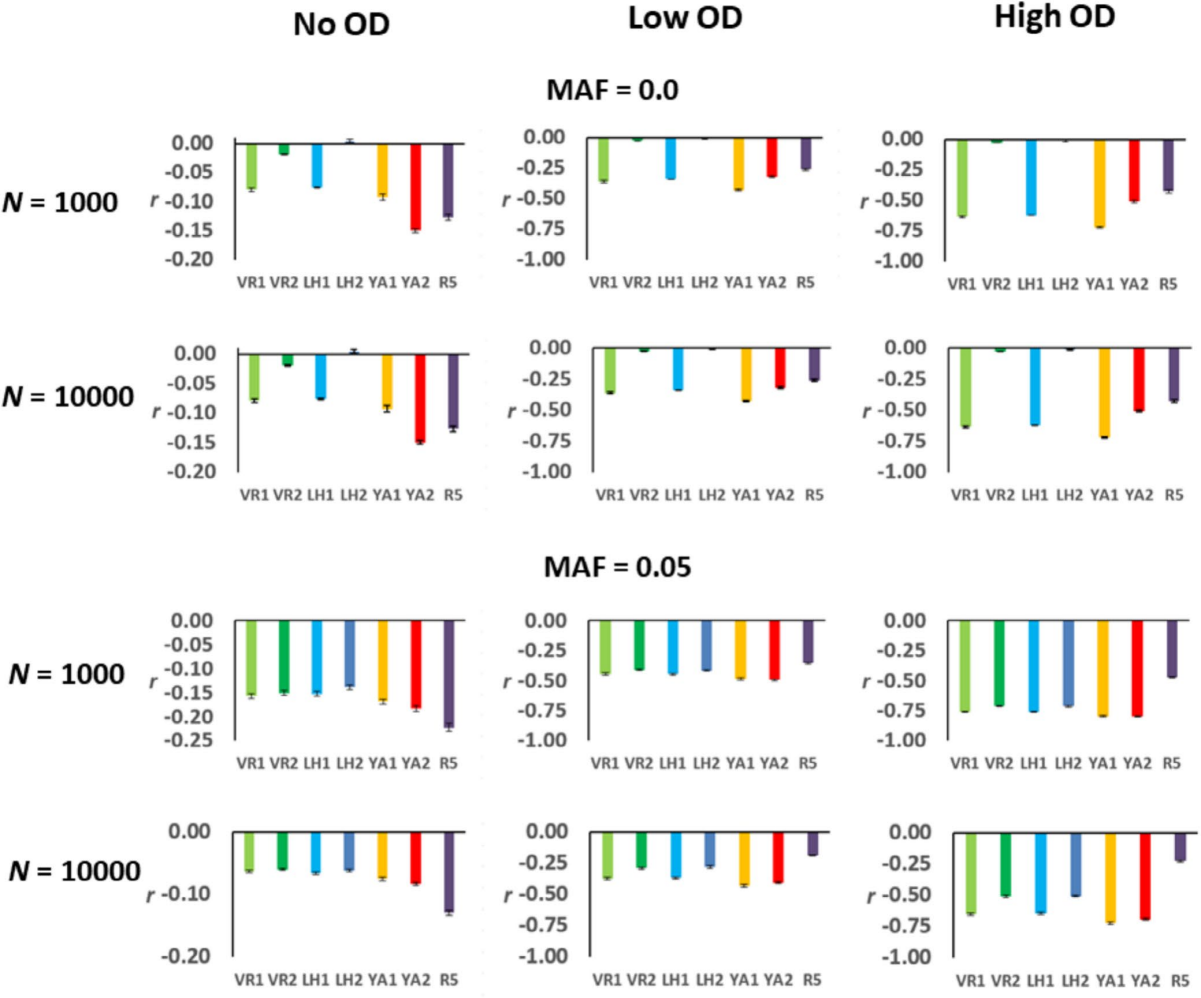
Correlation between the individual values of the genomic measures of the inbreeding coefficient (see text; only the subscripts of the estimators are shown for clarity) and the individual fitness. The scenarios assume partial recessive deleterious mutations without overdominance (No OD), or also adding a low rate (Low OD), or a high rate (High OD), of overdominant mutations. The results are the averages of simulations for 12 chromosomal segments and 20 replicates each, and bars indicate one standard error of the mean. See text for details on the simulation parameters.

Finally, Figure 3 shows the correlation between the individual *F* measures and the individual fitness. Without overdominance, *F*_*YA*2_ (with no MAF applied) and *F*_*R*5_ (with MAF applied) were the inbreeding measures showing the largest correlation with fitness, followed by *F*_*YA*1_. With overdominance, *F*_*YA*1_ (with no MAF applied) and both *F*_*YA*1_ and *F*_*YA*2_ (with MAF applied) were the measures showing the largest correlation with fitness.

## 4 Discussion

The increasing availability and decreasing cost of molecular markers have allowed the prediction of relationships between individuals in the absence of pedigree records, using a plethora of genomic measures of the inbreeding coefficient which can be used to estimate the rate of inbreeding depression (Lynch and Walsh 1998; Keller et al. 2011). A number of empirical and simulation studies have investigated the performance of these *F* measures to estimate inbreeding depression. However, all previous studies have ignored the possibility of overdominance, or balancing selection in general, as a source of inbreeding depression. There is a long‐standing debate in the literature about the major role of partial dominance versus the overdominance hypothesis of inbreeding depression, which has largely settled the prevailing consensus that the contribution of overdominance to inbreeding depression should be minor (Charlesworth and Charlesworth 1987; Crow 1999; Roff 2002; Charlesworth and Willis 2009; Yang et al. 2017). Nevertheless, several sources of evidence suggest that overdominance cannot be neglected (Hughes et al. 2006; Ayroles et al. 2009; Thurman and Barrett 2016), and even a small number of overdominant loci could have a large contribution to the inbreeding load (Kimura and Ohta 1971). In this work, we have evaluated the performance of the most popular measures of inbreeding from molecular markers to estimate inbreeding depression in the presence of overdominance. We found that estimates based on runs of homozygosity seem to be the most robust in general, whereas SNP‐by‐SNP estimators have a performance that depends more on the scenario and the pruning of low‐frequency alleles in the data. With overdominance, among these estimates, those from *F*_*YA*1_ were the most reliable if no MAF pruning was applied, whereas estimates from *F*_*YA*2_ were more accurate with MAF pruning.

The inbreeding load per locus generated by overdominance is expected to be much larger than that generated by dominance because the former is a function of the selection coefficient, whereas the latter is a function of the mutation rate (Kimura and Ohta 1971). We considered a set of mutational parameters for the dominance model that produced an inbreeding load close to one in the absence of overdominant loci. This estimate is approximately the average value observed in human populations (Bittles and Neel 1994; Lynch and Walsh 1998; Caballero 2020). For overdominance models, the increase in inbreeding load was up to about 2 (Low OD model) or 4–6 (High OD) (Tables 2 and S1). This is compatible with values observed empirically, which were between 2 and 3 for fecundity or viability in wild mammals and birds (O'Grady et al. 2006; Nietlisbach et al. 2019), or with larger estimates, even up to 11, obtained in other studies (e.g., Kruuk et al. 2002; Jensen et al. 2007; Grueber et al. 2011; Wolak et al. 2018; Trask et al. 2021; Fraimout et al. 2023). Thus, our simulated range of possible estimates of inbreeding depression is within the plethora of values observed in natural populations.

Overdominance represents a form of balancing selection in which alleles at overdominant loci are maintained in populations as polymorphisms at intermediate frequencies (Charlesworth 2006). With the asymmetrical model of overdominance considered, the expected equilibrium frequency of overdominant alleles would be 0.75. This frequency is attained in the simulation for the Low OD model (Tables 2 and S1) but is somewhat smaller for the High OD model. The reason is that because the rate of mutation for the high OD model is much larger, there will be some mutations that have not reached equilibrium and will segregate at lower frequencies, making the average frequency lower than 0.75. Selection on overdominant mutations produces a hitch‐hiking effect on neutral and deleterious mutations (Lu et al. 2006; Chun and Fay 2011; Hartfield and Otto 2011; Marsden et al. 2016), and this is reflected in the observed increase in allele frequency for neutral and deleterious mutations with increasing levels of overdominance (Tables 2 and S1). This increase in allele frequencies of loci closely linked to the overdominant locus is usually known as associative overdominance (Ohta and Kimura 1970; Bierne et al. 2000) and is also consistent with the observed increase in the inbreeding load arising from the deleterious and sterile alleles (Tables 2 and S1).

We have considered a model of pure overdominance at single loci. However, the interaction between different deleterious recessive mutations at closely linked loci in the repulsion phase can also generate pseudo‐overdominance (Frydenberg 1963; Ohta 1971), with results similar to those of true overdominance (Waller 2021; Abu‐Awad and Waller 2023). Pseudo‐overdominance is seen as a mechanism maintaining neutral diversity, especially in low recombination regions and small populations (Becher et al. 2020; Gilbert et al. 2020). In fact, some inbreeding depression that was initially thought to be caused by overdominance has been later suggested to be the result of pseudo‐overdominance (Carr and Dudash 2003; Charlesworth and Willis 2009; Bersabé et al. 2015; Draghi and Whitlock 2015; Becher et al. 2020; Gilbert et al. 2020). In addition, other sources of balancing selection can contribute as well to inbreeding depression. For example, selection in heterogeneous environments may generate scenarios with heterozygote advantage for fitness with similar outcomes to overdominance (Pamilo 1988; Glémin 2021). It may be difficult, though, to quantify which proportion of the estimated inbreeding depression is due to the effects of partially recessive deleterious mutations, and which is due to overdominance or other sources of balancing selection. However, these latter will generally produce an excess of genetic variance for fitness traits over that expected under deleterious mutation‐selection balance (Santos 1997; Mukai 1988; Charlesworth 2015; Sharp and Agrawal 2018), which can give a clue about the nature of the variation observed. In addition, several methods have been developed to detect signatures of balancing selection (Siewert and Voight 2017; Bitarello et al. 2018; Soni et al. 2022).

We considered different SNP‐by‐SNP measures of the inbreeding coefficient that are based on genetic drift derivations or on homozygosity derivations. Although they are formally analogous if allele frequencies of a putative base population are considered (Meuwissen et al. 2020; Caballero et al. 2021, 2022), they differ substantially if the allele frequencies of the current population are used in their calculations. The difference between the estimators with subscripts 1 and 2 is simply the way in which the averages are made, whether as the ratio of averages for loci or the average of ratios, respectively. The estimators *F*_*VR*1_ and *F*_*LH*1_ showed a generally better performance than *F*_*VR*2_ and *F*_*LH*2_, as already shown elsewhere in the absence of overdominance (Van Raden 2008; Villanueva et al. 2021; Caballero et al. 2021, 2022). With overdominance, the performance of the estimators *F*_*VR*2_ and *F*_*LH*2_ does not improve, although the pruning of low‐frequency alleles makes them more accurate (Figures 1 and 2).

The estimator based on the correlation between uniting gametes (*F*_*YA*2_) was shown to be rather accurate in estimating ID without overdominance and to have the highest correlation with fitness in this scenario, as shown in previous simulation studies (Yengo et al. 2017; Caballero et al. 2021, 2022). This estimator was also shown to have the largest empirical correlation with homozygous mutation load measured with homozygous deleterious missense variants (Alemu et al. 2021). However, in the presence of overdominant loci, *F*_*YA*2_ becomes highly biased upward and it is no longer the measure with a higher correlation with fitness (Figures 1, 2, 3). This was confirmed by considering a scenario where only overdominant mutations occur (Figure S3). The general effect of overdominance is to increase the estimated ID, and this occurs for all molecular measures of inbreeding (Figure 1). However, the effect is more pronounced for the estimates from *F*_*YA*2_. This estimator is known to give a large weight to low‐frequency alleles because homozygous genotypes are weighted by the inverse of their allele frequencies (Yang et al. 2011). In fact, *F*_*YA*2_ can also be obtained as $F_{\mathrm{YA}2}=\left(\sum_{k=1}^{S}\delta_{k}/S\right)-1$ (Keller et al. 2011), where *δ* equals 1/*p*_*k*_ for homozygotes of the minor allele, 1/(1—*p*_*k*_) for homozygotes of the major allele, and zero for the heterozygotes, which explicitly shows the larger weight given to rare rather than to common alleles. Overdominance increases the frequency of neutral alleles (Table 2) and this effect will be larger for individuals with more overdominant loci and higher fitness. For these individuals, *δ* for minor allele homozygotes will be reduced and so the estimated *F*_*YA*2_, implying an overestimation of ID with this inbreeding measure. This effect will be smaller for *F*_*YA*1_ because, in this case, the estimates of inbreeding are based on averages across loci (cf. the equations of *F*_*YA*1_ and *F*_*YA*2_ in the Methods section). In fact, Lavanchy et al. (2024) showed that *F*_*YA*1_, which gives more weight to common alleles, is more useful than *F*_*YA*2_ for the estimation of ID in subdivided populations. Accordingly, with overdominance, the correlation between fitness and *F*_*YA*1_ becomes larger than that with *F*_*YA*2_ (Figures 3 and S3). By applying an MAF pruning to the data, the impact of rare alleles on *F*_*YA*2_ estimates will be diminished, and the overestimation of ID from this molecular inbreeding measure is reduced (Figures 1 and 2).

As far as we know, only one previous study has considered the impact of overdominance for a two‐locus model comparing the estimates of inbreeding depression estimated from pedigree inbreeding and molecular autozygosity for the two loci (Curik et al. 2001). This study showed that under overdominance, the estimated inbreeding depression with pedigree inbreeding was lower than with true autozygosity. Thus, the use of pedigree inbreeding could lead to an underestimation of the inbreeding load from overdominant loci. In the present study, we have not considered pedigree inbreeding. The correlation between this measure of inbreeding and the molecular measures considered in this paper is generally intermediate, as obtained from simulations and empirical data, with a great variation. For instance, the average empirical correlation between pedigree inbreeding and *F*_*YA*2_ is 0.42 as gathered from six studies (see Caballero et al. 2022), but with a wide range between 0.15 and 0.72. Similar intermediate average correlations and wide ranges are found between pedigree inbreeding and other measures of molecular inbreeding. For many measures of inbreeding evaluated in the present work, the estimates of inbreeding depression with overdominance were underestimates, in agreement with the results of Curik et al. (2001).

Estimates based on ROH seem to be the most robust for all scenarios, either with or without overdominance and with MAF pruning or not (Figures 1, 2 and S3). The estimates are generally more accurate when large ROH are considered (Figure S2), in agreement with previous studies in the absence of overdominance (Caballero et al. 2021, 2022; Pérez‐Pereira, Quesada, et al. 2023). It is well known that the measurements of inbreeding obtained from ROH largely depend on the criterion used to define ROH, particularly their length. Because recombination breaks long chromosomal segments, longer ROH are expected when there is a recent common ancestor, and shorter ROH when the common ancestor is more distant (Broman and Weber 1999). In other words, the longer the homozygous segments, the more recent the inbreeding, and short ROH denotes also ancient inbreeding, but they could imply not only identity by descent fragments but also some identity by chance (Ferenčaković et al. 2013, 2017; Pryce et al. 2014; Marras et al. 2015). An overestimated inbreeding from short ROH segments would imply an underestimation of ID. In fact, studies comparing ROH of different lengths have found that fragments smaller than 4 Mb are less likely to be due to pedigree‐based inbreeding than larger ones (Purfield et al. 2012; Ferenčaković et al. 2013). Recent studies have also shown that inbreeding depression is more associated with long homozygous‐by‐descent (Naji et al. 2024) or long ROH genomic fragments (Makanjuola et al. 2020; Stoffel et al. 2021) than with short ones, in agreement with the observation that longer (more recent) ROH are enriched in deleterious mutations (Szpiech et al. 2013).

In summary, our simulation results indicate that estimates of inbreeding depression from *F*_*ROH*_ are the most robust to the presence of overdominant loci contributing to the inbreeding load, particularly if ROH is long. Estimates of inbreeding depression from SNP‐by‐SNP‐based *F* measures can be highly biased depending on the scenarios and the pruning of low‐frequency alleles, but the estimators based on the correlations of uniting gametes (*F*_*YA*1_, *F*_*YA*2_) are generally the most reliable.

## Conflicts of Interest

The authors declare no conflicts of interest.

## Supporting information


Appendix S1.

## Data Availability

Simulation codes and scripts are available at Github address: https://github.com/ines‐gonzalezcastellano/inbreeding‐depression‐with‐overdominance.
