# Supplementary material for: Estimation of Inbreeding Depression From Overdominant Loci Using Molecular Markers
Source: Evol Appl. 2025 Mar 13;18(3):e70085. doi: 10.1111/eva.70085 (PMC11906488; doi:10.1111/eva.70085)
Supplement: Supplementary file 1 — Appendix S1. [file EVA-18-e70085-s001.docx]

**SUPPLEMENTARY MATERIAL**

**Estimation of inbreeding depression from overdominant loci using molecular markers**

**Table S1.** Average number of segregating loci and mean allelic frequency of neutral, deleterious, sterile, and overdominant mutations, and inbreeding load attributable to deleterious (*B_del_*), sterile (*B_ste_*), overdominant (*B_o_*), or all (*B*) mutations. Results refer to a population of size *N* = 10,000 for scenarios with partial recessive deleterious mutations without overdominance (No OD), or also adding a low rate (Low OD), or a high rate (High OD), of overdominant mutations. The results are the averages of simulations for 12 chromosomal segments and 20 replicates each, and its corresponding standard errors.

| **Model** | **Neutral** | **Deleterious** | **Sterile** | **Overdominant** |
| --- | --- | --- | --- | --- |
| **Num. of SNPs** |  |  |  |  |
| noOD | 38930.7 ± 456.1 | 5228.20 ± 61.85 | 295.42 ± 3.58 | - |
| lowOD | 35684.0 ± 419.0 | 5061.87 ± 58.72 | 291.95 ± 3.56 | 174.75 ± 2.22 |
| highOD | 29238.9 ± 329.4 | 4488.75 ± 50.95 | 259.46 ± 3.14 | 748.14 ± 8.84 |
| **Freq. of SNPs** |  |  |  |  |
| noOD | 0.029 ± 0.000 | 0.006 ± 0.000 | 0.001 ± 0.000 | - |
| lowOD | 0.031 ± 0.000 | 0.007 ± 0.000 | 0.001 ± 0.000 | 0.710 ± 0.001 |
| highOD | 0.035 ± 0.001 | 0.008 ± 0.000 | 0.001 ± 0.000 | 0.697 ± 0.001 |
| **Inbreeding load** | ***B*** | ***B_del_*** | ***B_ste_*** | ***B_o_*** |
| noOD | 0.886 ± 0.011 | 0.541 ± 0.006 | 0.345 ± 0.005 | - |
| lowOD | 2.136 ± 0.026 | 0.542 ± 0.007 | 0.352 ± 0.005 | 1.242 ± 0.016 |
| highOD | 6.286 ± 0.074 | 0.555 ± 0.008 | 0.343 ± 0.005 | 5.387 ± 0.063 |

**Table S2.** Average estimates of the inbreeding coefficient obtained from neutral loci for a population of size *N* = 1,000 or 10,000 individuals assuming partial recessive deleterious mutations without overdominance (No OD), or also adding a low rate (Low OD) or a high rate (High OD) of overdominant mutations. The results are the averages of simulations for 12 chromosomal segments and 20 replicates each, and its corresponding standard errors (se).

|  | ***F_VR1_*** | ***F_VR2_*** | ***F_LH1_*** | ***F_LH2_*** | ***F_YA1_*** | ***F_YA2_*** | ***F_NJ_*** | ***F_R01_*** | ***F_R1_*** | ***F_R5_*** |
| --- | --- | --- | --- | --- | --- | --- | --- | --- | --- | --- |
| ***N* = 1000** |  |  |  |  |  |  |  |  |  |  |
| **No OD** | **-0.000480** | **-0.000488** | **-0.000480** | **-0.000488** | **-0.000480** | **-0.000488** | **0.888204** | **0.220026** | **0.076292** | **0.014233** |
| se | 0.000138 | 0.000073 | 0.000138 | 0.000073 | 0.000138 | 0.000073 | 0.000205 | 0.000962 | 0.000606 | 0.000266 |
| **Low OD** | **-0.000332** | **-0.000471** | **-0.000332** | **-0.000471** | **-0.000332** | **-0.000471** | **0.877126** | **0.216691** | **0.076703** | **0.014378** |
| se | 0.000224 | 0.000091 | 0.000224 | 0.000091 | 0.000224 | 0.000091 | 0.000427 | 0.000869 | 0.000619 | 0.000284 |
| **High OD** | **-0.000268** | **-0.000408** | **-0.000268** | **-0.000408** | **-0.000268** | **-0.000408** | **0.853166** | **0.208102** | **0.084602** | **0.016890** |
| se | 0.000289 | 0.000122 | 0.000289 | 0.000122 | 0.000289 | 0.000122 | 0.000670 | 0.000775 | 0.000860 | 0.000354 |
|  |  |  |  |  |  |  |  |  |  |  |
| ***N* = 10000** |  |  |  |  |  |  |  |  |  |  |
| **No OD** | **-0.000011** | **-0.000045** | **-0.000011** | **-0.000045** | **-0.000011** | **-0.000045** | **0.954550** | **0.450850** | **0.042367** | **0.004541** |
| se | 0.000023 | 0.000009 | 0.000023 | 0.000009 | 0.000023 | 0.000009 | 0.000043 | 0.001064 | 0.001103 | 0.000425 |
| **Low OD** | **-0.000059** | **-0.000052** | **-0.000059** | **-0.000052** | **-0.000059** | **-0.000052** | **0.959806** | **0.572560** | **0.129964** | **0.011985** |
| se | 0.000027 | 0.000008 | 0.000027 | 0.000008 | 0.000027 | 0.000008 | 0.000064 | 0.001248 | 0.001690 | 0.001031 |
| **High OD** | **-0.000028** | **-0.000055** | **-0.000028** | **-0.000055** | **-0.000028** | **-0.000055** | **0.960746** | **0.624190** | **0.213224** | **0.017945** |
| se | 0.000037 | 0.000008 | 0.000037 | 0.000008 | 0.000037 | 0.000008 | 0.000195 | 0.002025 | 0.001518 | 0.001452 |

**Note:** The estimates of inbreeding from ROH are among those having the lowest relative standard errors, so they may provide useful comparisons. Mean estimates from ROH < 0.1 Mb (*F_R0_*_1_) were very large, suggesting that many small fragments may not be identical by descent (IBD). Note that the whole population was analysed in each case so that more numerous smaller ROH were found in the *N* =10,000 population than in the *N* = 1,000 population. When looking at mean estimates of inbreeding coefficient for larger ROH (*F_R_*_1_ or *F_R_*_5_, more likely to involve IBD), then the mean inbreeding coefficient without overdominance was higher for the *N* = 1,000 population (0.076 for *F_R_*_1_ and 0.014 for *F_R_*_5_) than for the *N* =10,000 population (0.042 and 0.0045, respectively), as expected. From *F_R_*_1_ and *F_R_*_5_ estimates of inbreeding, it can be deduced that the observed effect of overdominance was an increase in the estimated inbreeding coefficient.

**Figure S1.** Chromosomal segments of the human genome considered to obtain the map of recombination frequencies used in the simulations.


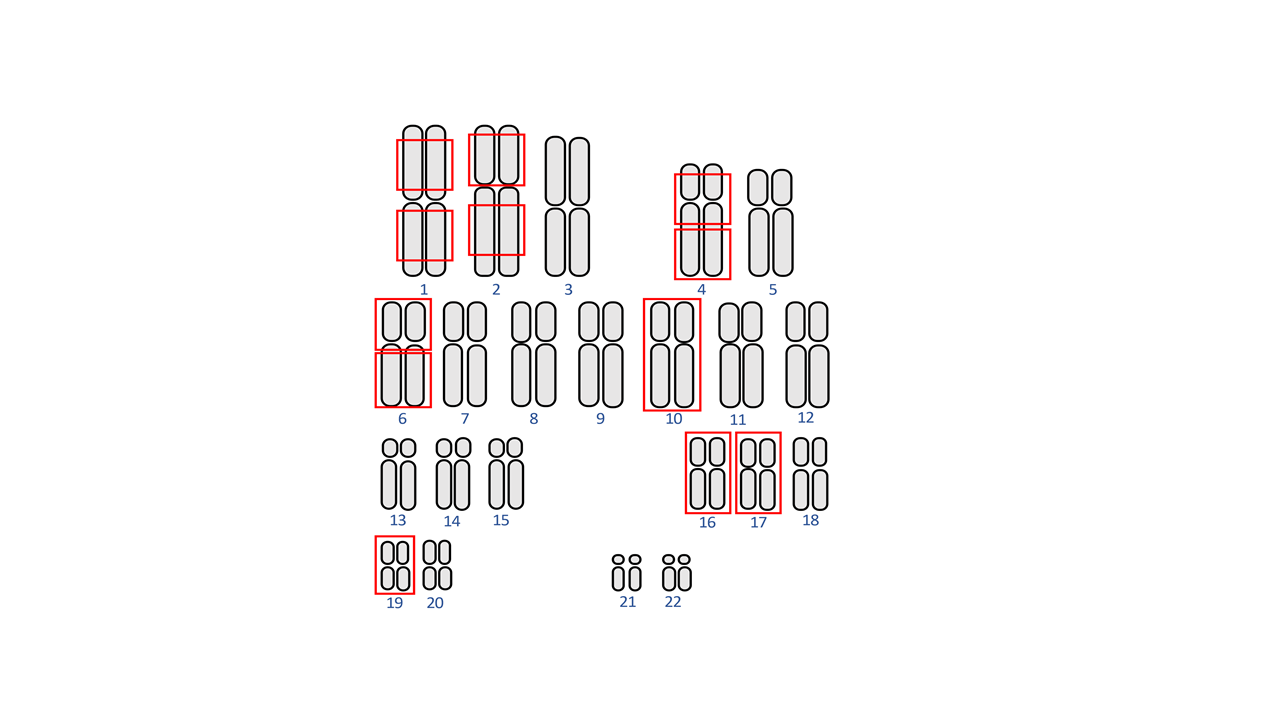


**Figure S2**. Root mean square error (RMSE) of estimates of inbreeding depression obtained from runs of homozygosity (ROH) segments with lengths larger than 0.1 Mb (R01), 1 Mb (R1) or 5 Mb (R5). Simulations assume a population size of *N* = 1,000 or *N* = 10,000 individuals, with no MAF pruning, partial recessive deleterious mutations without overdominance (No OD), or also adding a low rate (Low OD), or a high rate (High OD), of overdominant mutations. The results are the averages of simulations for 12 chromosomal segments and 20 replicates each, and bars indicate one standard error of the mean.

**
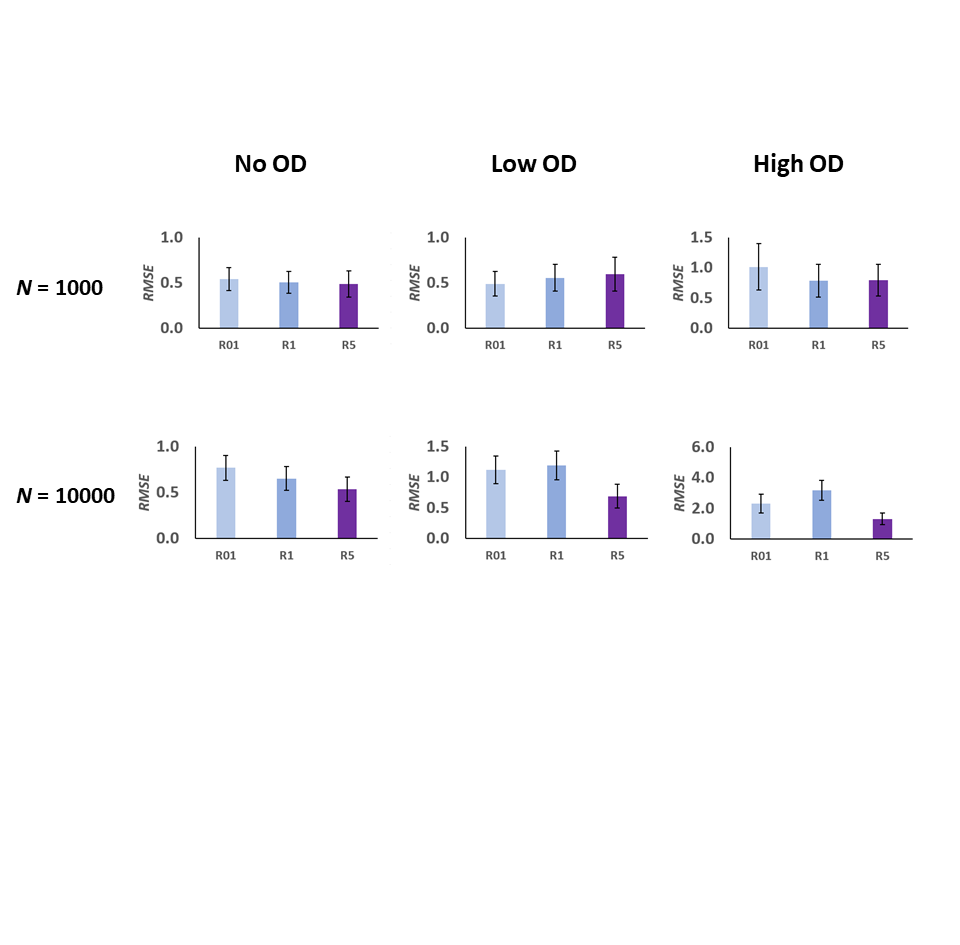
**

**Figure S3**. Simulation assuming only overdominant mutations, appearing at a low rate (Low OD) or at high rate (High OD). **(a)** Proportional deviation of the estimates of inbreeding depression (ID) obtained with different measures of the inbreeding coefficient with marker data (see main text), with respect to the true simulated ID value. The dot is the mean deviation and the bar indicates the 95% of the distribution of simulated replicates. **(b)** Root mean square error (RMSE) of estimates of inbreeding depression. Bars indicate one standard error of the mean. **(c)** Correlation between the individual values of the genomic measures of the inbreeding coefficient (see text) and the individual fitness. Bars indicate one standard error of the mean. Simulations assume a population size of *N* = 1000 and no MAF pruning The results are the averages of 20 simulated replicates.

**
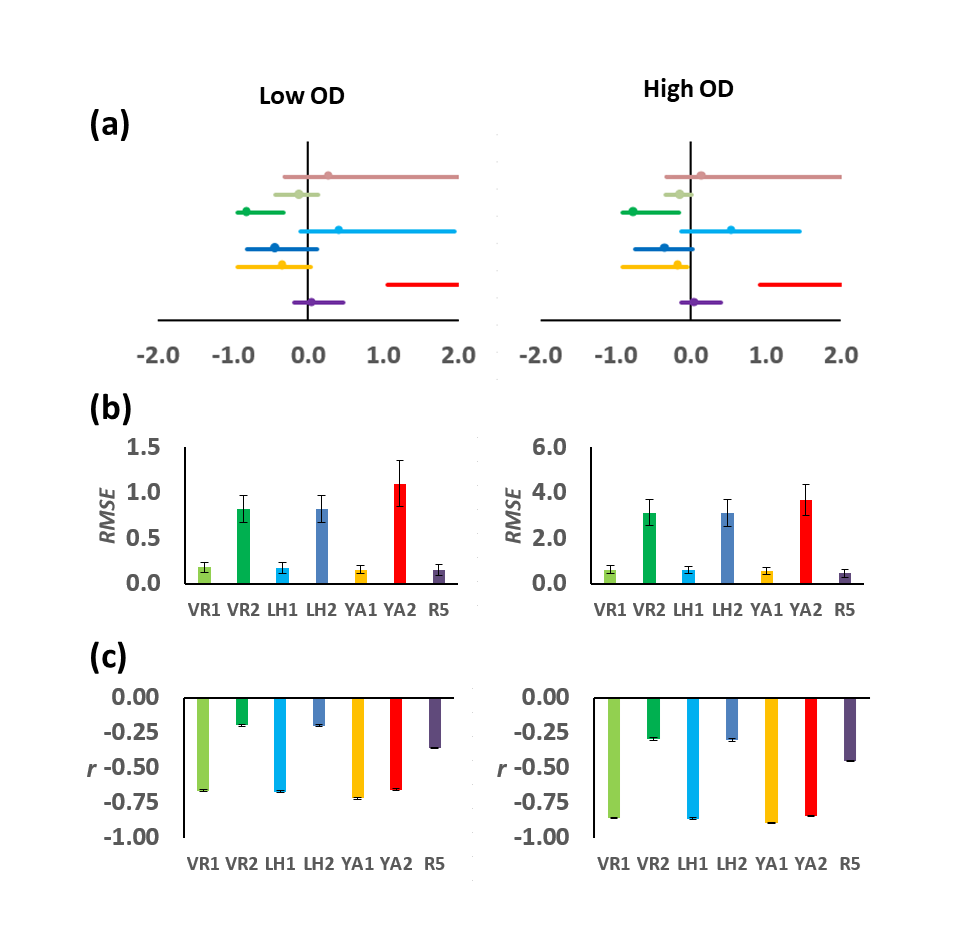
**

**Relationship between the estimates of ID from** ***F_LH_*_1_ and *F_NJ_***

The measures of inbreeding *F_NJ_* and *F_LH_*_1_ have a correlation of one, but differ in the estimates of ID they provide because of their different scales. The estimate *F_NJ_* measures the observed proportion of homozygous SNPs, i.e. *F_NJ_* = O[*H*] / *S*, where O[*H*] is the observed number of homozygous SNPs in each individual and *S* is the number of SNPs. On the other hand, the estimator *F_LH_*_1_ measures the difference between the observed and expected numbers of homozygous SNPs, relative to the expected number of heterozygous SNPs, i.e., *F_LH_*_1_ = (O[*H*] – E[*H*]) / (*S* – E[*H*]). Thus, the estimates of ID from each of them, obtained as the regression of individual fitness values (*W*) on the inbreeding estimates, are:

$$b_{W.F_{NJ}}=\frac{cov\left( W,\frac{\text{O}[H]}{S} \right)}{var\left( \frac{\text{O}[H]}{S} \right)}=\frac{\frac{1}{S}cov\left( W,\text{O}[H] \right)}{\frac{1}{S^{2}}var\left( \text{O}[H] \right)}=S\frac{cov\left( W,\text{O}[H] \right)}{var\left( \text{O}[H] \right)}$$

and

$$b_{W.F_{LH1}}=\frac{cov\left( W,\frac{(\text{O}\left[ H \right]-\text{E}\left[ H \right])}{(S-\text{E}\left[ H \right])} \right)}{var\left( \frac{(\text{O}\left[ H \right]-\text{E}\left[ H \right])}{(S-\text{E}\left[ H \right])} \right)}=\frac{\frac{1}{(S-\text{E}\left[ H \right])}cov\left( W,\text{O}[H] \right)}{\frac{1}{{(S-\text{E}\left[ H \right])}^{2}}var\left( \text{O}[H] \right)}=(S-\text{E}\left[ H \right])\frac{cov\left( W,\text{O}[H] \right)}{var\left( \text{O}[H] \right)}$$

because both *S* and E[*H*] are invariable for a given sample and set of SNPs.

Therefore,

$$b_{W.F_{LH1}}=\frac{S-\text{E}\left[ H \right]}{S}b_{W.F_{NJ}}$$

That is, the estimate of ID from *F_LH_*_1_ equals that from *F_NJ_* multiplied by the expected frequency of heterozygotes.

For example, for a particular simulation in the scenario with *N* = 1,000 individuals, no MAF pruning and high overdominance, the estimate of ID using *F_NJ_* was 26.64, whereas that using *F_LH_*_1_ was 3.73, and the expected frequency of heterozygotes in the population was 0.14.
